# Guiding Maxillary Left Central Incisor to Occlusion and Late Formation of a Supernumerary Tooth in the Upper Left Premolar Region

**DOI:** 10.1155/2021/6622641

**Published:** 2021-01-30

**Authors:** I. A. Alnaqbi, A. O. Mageet

**Affiliations:** ^1^Specialist Orthodontist, Ministry of Health & Prevention, Sharjah, UAE; ^2^Orthodontic Resident, Hamdan Bin Mohammed College of Dental Medicine, MBRU, Dubai, UAE; ^3^Associate Professor of Orthodontics, Faculty of Dentistry, Ajman University and a Member of the Center of Medical and Bio-allied Health Sciences, Ajman University, Ajman, UAE; ^4^Associate Prof. of Orthodontics, Hamdan Bin Mohammed College of Dental Medicine, MBRU, Dubai, UAE

## Abstract

Hyperdontia or supernumerary teeth are the erupted or impacted teeth, which develop in addition to the regular dental series and might cause many occlusal problems. This article sheds light on a case of impacted maxillary left central incisor (21) due to a mesiodens supernumerary tooth and a late development of another supernumerary tooth in the upper left premolar area at the end of orthodontic treatment. O.A. is an 11-year, eight-month-old male, African patient presented to the orthodontic clinic with a chief complaint “My upper front tooth did not erupt although the dentist attached a wire to pull it with the help of a neighbouring tooth.” Clinically, he is medically fit and healthy, presented with class II division I malocclusion on skeletal II base; mild space discrepancy in the upper and lower dental arches; impacted 21; increased overjet; reduced overbite; localized bilateral posterior crossbite in relation to tooth number 16, 15, 25, and 26; and lower centreline shift to the left. Radiographically, lateral cephalometric radiograph confirms the skeletal relationship, whereas dental panoramic tomography (DPT) shows impacted 21 and the presence of all permanent teeth. The treatment plan consists of comprehensive orthodontic treatment using preadjusted edgewise metallic bracket, Roth prescription 0.022^″^ × 0.028^″^ slot and an active transpalatal arch (TPA) with palatal arms. Retention regimen comprises of upper and lower bonded retainers from canine to canine and vacuum-formed retainers (VFRs) for both dental arches.

## 1. Introduction

Supernumerary teeth could be found in many parts of the human body such as the ovary, nasal cavity, or hard palate; however, they are normally seen in the dental arches [1–3] with a higher incidence in the maxillary anterior region [4]. Development of these teeth can be in one or both dental arches, in a single or multiple forms, and their location could be of unilateral or bilateral [5]. The prevalence of supernumeraries in primary dentition was found to be 0.3-0.8% whereas it was 1.5-3.5% in permanent dentition [6, 7]. Their presence varies among populations which is more in Asians [3, 8], and they are in males more than females [9]. The aetiology of supernumerary teeth is multifactorial [10], with different theories suggested dichotomy of tooth bud, hyperactivity of dental lamina [5, 6, 11], and genetic influences [12].

The frequency of supernumerary premolars was as high as 0.64% (7 in 1,100) as seen in orthodontic population, and the age range of the patients when detecting the supernumerary premolars was between 11 and 16 years [13]. Several syndromes have been associated with supernumeraries such as Down's syndrome, cleidocranial dysplasia, cleft lip and palate, Gardner syndrome, Ellis-van Creveld syndrome, Zimmermann-Laband syndrome, or Noonan syndrome [14]. The clinical signs of supernumerary teeth are reflected by spacing especially midline diastema, failure of adjacent teeth to erupt mainly upper incisors, and local crowding or irregularity [15].

## 2. Case Presentation

An 11-year, eight-month old Sudanese boy attended to the orthodontic clinic with a chief complaint that “My upper front tooth did not erupt although the dentist attached a wire to pull it with the help of a neighbouring tooth.” Medically, he was fit and healthy and had no reported habits. According to his parents, he had a history of a supernumerary tooth in the upper left central incisor, but unfortunately, photos are not available.

### 2.1. Clinical Examination: Extraoral Features

In the transversal plane, the five-fifths are averagely equal with no facial asymmetry; vertically, the lower facial height is slightly increased, while the sagittal plane shows mild convex facial profile. The soft tissue examination shows average nasolabial angle, competent lips, 90% of upper incisors on smiling, small buccal corridors, and an average mentolabial fold. The TMJ assessment revealed no clicking, no crepitus, no deviation, or limitation in mouth opening.

### 2.2. Clinical Examination: Intraoral Features

**Figure 1:**
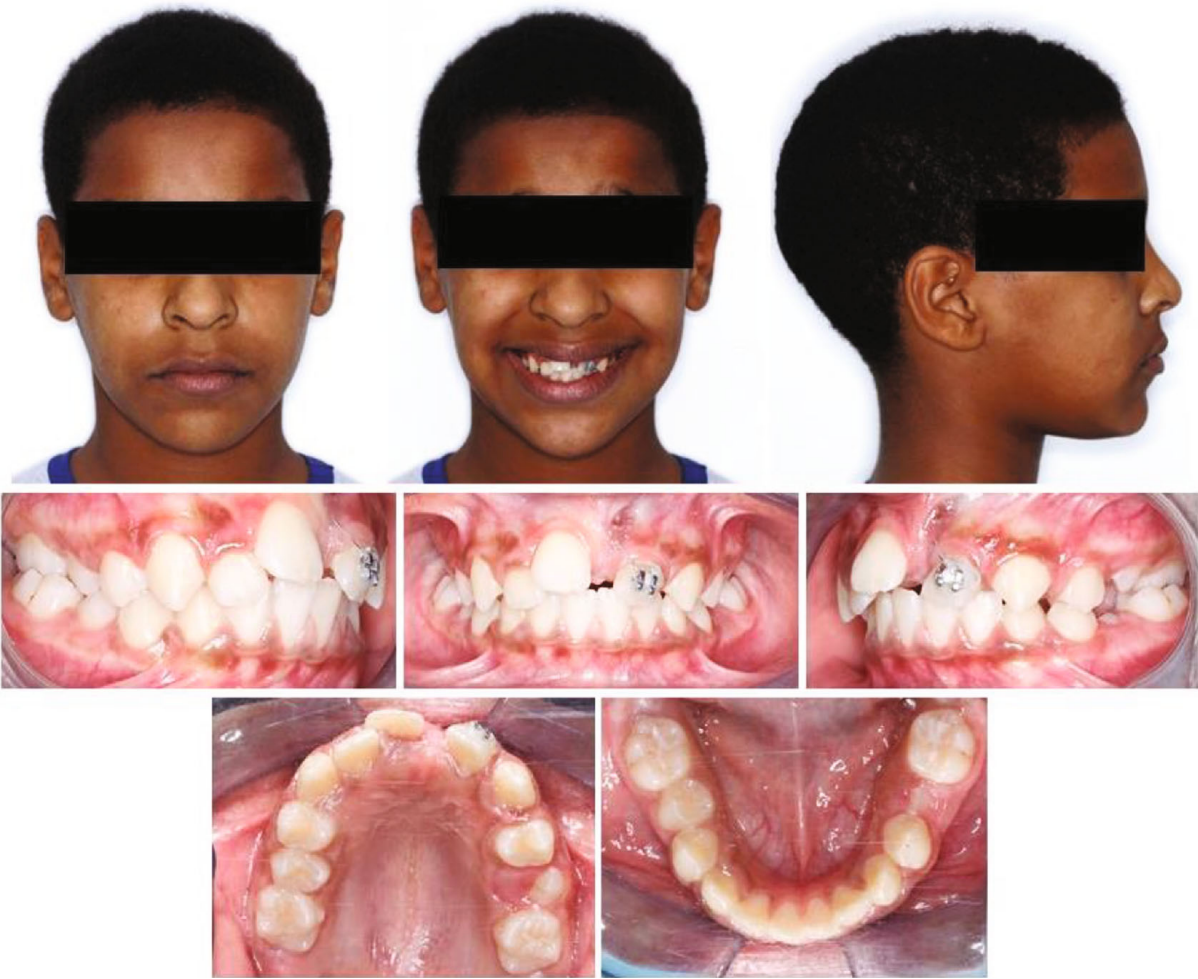
Pretreatment extra- and intraoral photographs.

The patient presented with adequate oral hygiene impacted 21 and mild space discrepancy in both dental arches. The Bolton overall ratio was 89.58% whereas the anterior ratio was 80.21%. Occlusal assessment exhibited a class II/1 incisor relationship; class II canine relationship; class I molar relationship; increased overjet of 5 mm; reduced overbite; localized crossbite of tooth number 16, 15, 25, and 26; and mild shift of the lower centreline to the left (metallic ligature connected between tooth nos. 21 and 22) (Figure 1).

### 2.3. Radiographic Features

**Figure 2:**
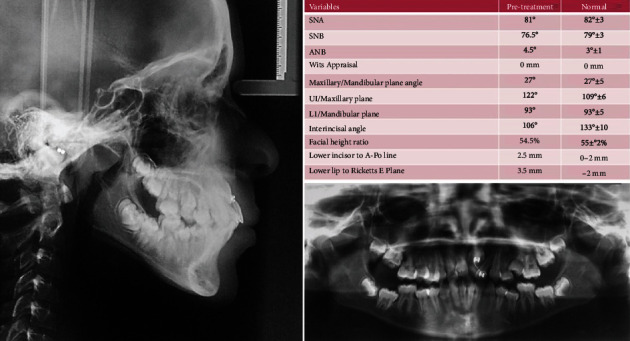
Pretreatment radiographs and analysis.

The DPT shows no pathologies or root resorption, presence of all permanent teeth, and an impacted tooth number 21. Analysis of the lateral cephalometric radiograph confirms mild class II skeletal relationship, increased lower anterior facial height, proclined upper incisors relative to the maxillary plane, and convex soft tissue profile (Figure 2).

## 3. Treatment Plan

### 3.1. Aim of the Treatment

The treatment was aimed at improving the oral hygiene, aligning tooth number 21 into the dental arch, achieving a class I incisor and canine relationships, correcting posterior crossbite, relieving crowding in both dental arches, achieving normal overjet and overbite, improving incisor display on smiling, correcting lower centreline shift, and long-term stability and retention.

### 3.2. Treatment Strategy

Treatment strategies include improving oral hygiene, comprehensive orthodontic treatment with upper and lower fixed appliance “pre-adjusted edgewise metallic bracket, and Roth prescription with 0.022^″^ × 0.028^″^ slot”; surgical exposure of tooth number 21 and aligning it using a loop-formed metallic ligature, correcting posterior crossbite employing active TPA with palatal arms; and relieving dental crowding through interdental striping.

### 3.3. Treatment Duration

Two years of active treatment.

### 3.4. Retention Protocol

The retention protocol comprises of an upper and lower bonded retainer from canine to canine and nighttime vacuum-formed retainer (VFR) for both dental arches.

## 4. Treatment Progress

**Figure 3:**
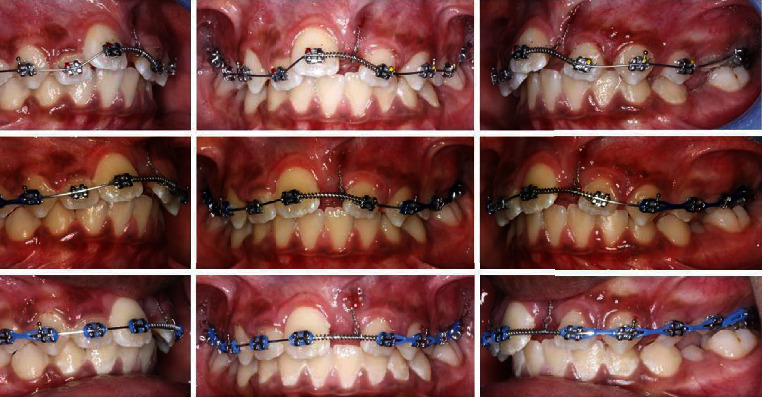
Initial stages.

Initially, TPA is cemented on upper first molars, upper teeth are bonded, and 0.014^″^ nickel-titanium (Ni-Ti) archwire was placed with a passive coil between 11 and 22 to maintain the space for 21. The metallic ligature that extended from the impacted tooth is just ligated against the base archwire. In the second visit, the upper archwire changed to 0.016^″^ Ni-Ti, followed in the next visit by an upper archwire 0.016^″^ × 0.022^″^ Ni-Ti and power chain connected the canine to the first molar on each side (Figure 3).

**Figure 4:**
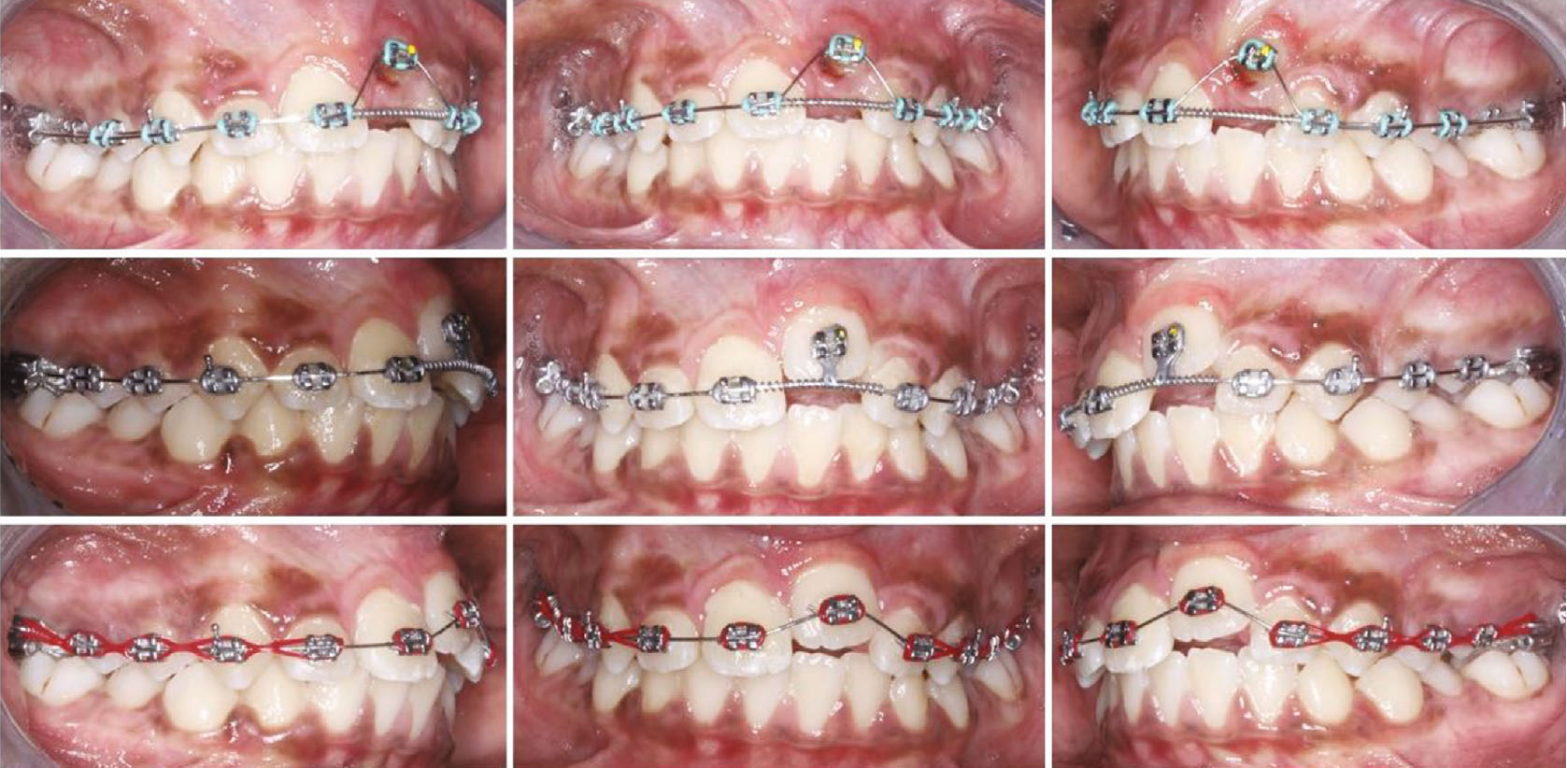
Treatment progress.

After the alignment of upper teeth, piggyback 0.012^″^ Ni-Ti archwire was used along with 0.018^″^ × 0.025^″^ Stainless Steel (SS) base archwire to align tooth number 21. Once the tooth number 21 approaches the dental arch, the wire mentioned above was replaced by a 0.014^″^ Ni-Ti archwire to achieve full alignment (Figure 4).

In the next visits, treatment started for the lower dental arch, and manual interdental stripping was performed for lower anterior teeth to relieve the crowding.

**Figure 5:**
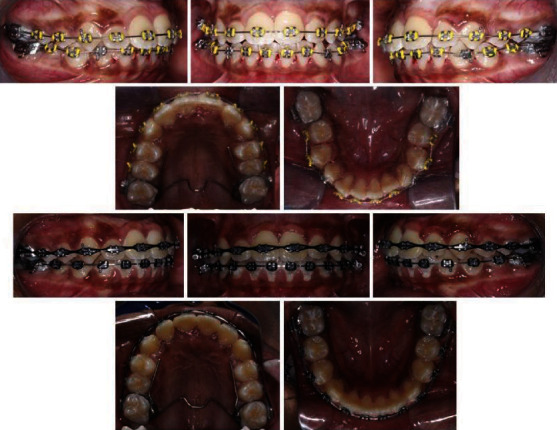
Bonding of the lower arch and finishing.

Meanwhile, the TPA was removed, activated, and recemented to correct the buccal crossbite (Figure 5).

Toward the end of orthodontic treatment, DPT and lateral cephalometric radiographs were taken to evaluate the progress of the treatment.

**Figure 6:**
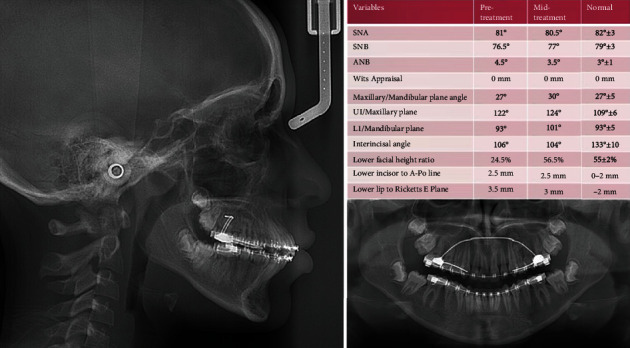
Midtreatment radiographs and analysis.

Radiological examination of the DPT showed the radiopaque area in the upper left premolar area (Figure 6).

The consultation was sought from the oral and maxillofacial radiologist who advised for Cone Beam Computed Tomography (CBCT) for further examination.

**Figure 7:**
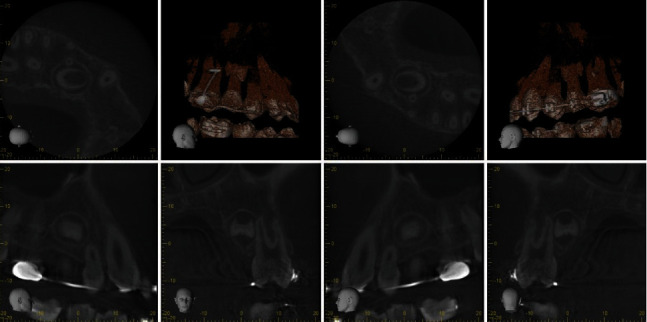
CBCT shows new supernumerary tooth developing in the upper left quadrant.

CBCT revealed the presence of a newly developed supernumerary tooth in the palatal side between the upper left first and second premolars (Figure 7).

The case was discussed with an oral and maxillofacial surgeon who advised for surgical removal of this tooth to eliminate any possibility of future complications.

**Figure 8:**
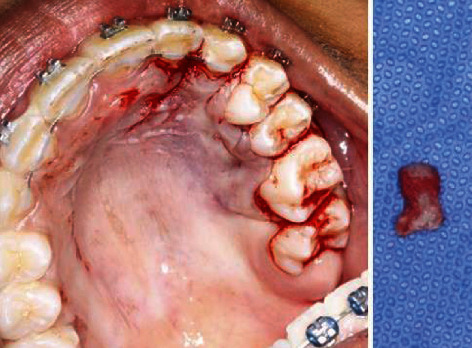
Removal of the supernumerary between the teeth 24 and 25.

Patient was transferred to the surgery department for surgical removal of the supernumerary tooth (Figure 8).

The patient followed for two months after the surgery to evaluate the healing process and allow further settling of the occlusion.

**Figure 9:**
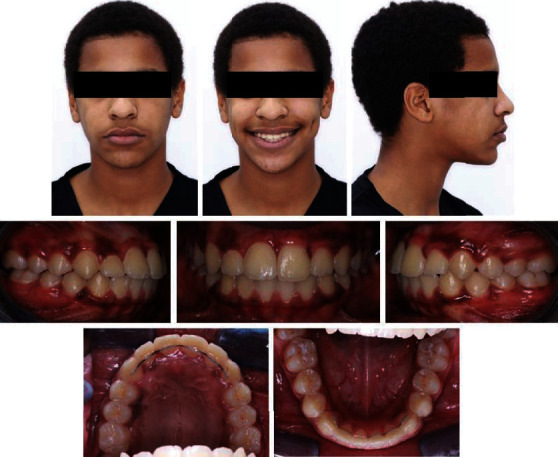
Posttreatment extra- and intraoral photographs.

After that, both arches were debonded and posttreatment instructions given to the patient (Figure 9).

### 4.1. Retention Regimen

The retention regimen comprises of fixed upper and lower retainers from canine to canine in addition to upper and lower vacuum-formed retainers (VFRs) for both dental arches. The follow-up will be for 3 years as follows:

- 1^st^ year: 4 visits (once every 3 months)—check for oral hygiene and the bonded retainers; the VFRs will be worn at nighttime only
- 2^nd^ year: 2 visits (once every 6 months)—reduce retainer wear (3 nights/week for 6 months, 2 nights/week for the next visit)
- 3^rd^ year: 2 visits (once every 6 months)—reduce retainer wear (one night/week for 6 months), check once/week for the next 6 months). Stop the VFRs

## 5. Discussion

The development of supernumerary teeth in the dental arch is not a rare phenomenon, and the incidence, in a decreasing order, is maxillary lateral incisors, mesiodens, maxillary central incisors, and premolars [16]. According to their morphology, supernumerary teeth can be classified into odontomes, rudimentary, tuberculate, and supplemental type [5]. Presence of these teeth in the dental arch might cause many dental problems and complicate orthodontic treatment [16]. One of these dental problems is impaction of the adjacent teeth as it occurred in our case. Early detection and management of extra teeth in the dental arch are a critical factor in minimizing the subsequent dental problems and therefore facilitate orthodontic treatment [17]. The general dental practitioner should learn when to refer the impacted teeth cases to the orthodontist, as these cases require extra tools for diagnosis and more experience in diagnosis and management. One of these tools is CBCT, which provide a precise location for the impacted teeth, determine the accurate position related to adjacent structures, and therefore enable the surgeon for correct surgical removal [17, 18].

## 6. Conclusion

Early detection of impacted supernumerary teeth is essential in achieving good results. General dental practitioners are advised to seek a consultation or refer cases that involve impacted teeth to the orthodontist for better diagnosis and correct management.

In our case, we decided to align the impacted upper left central incisor utilizing a loop-formed metallic ligature and surgical removal of impacted supernumerary tooth in the premolar area to avoid any future complications. Bonded retainers, VFRs, and class I occlusion will provide long-term stability and retention.
